# Comparative Transcriptome Analyses of Resistant and Susceptible Near-Isogenic Wheat Lines following Inoculation with *Blumeria graminis* f. sp. *tritici*

**DOI:** 10.1155/2017/7305684

**Published:** 2017-05-01

**Authors:** Piyi Xing, Xueying Zhang, Yinguang Bao, Yuhai Wang, Honggang Wang, Xingfeng Li

**Affiliations:** ^1^State Key Laboratory of Crop Biology, Shandong Agricultural University, Taian, Shandong, China; ^2^Agronomy College, Shandong Agricultural University, Taian, Shandong, China; ^3^College of Life Science, Zaozhuang University, Zaozhuang, Shandong, China

## Abstract

Powdery mildew is one of the most important diseases of wheat. In this study, the leaf RNA samples of wheat NILs carrying powdery mildew resistant and susceptible Pm2 alleles (L031 and Chancellor) and its F1 hybrid at two time points (16 h and 96 h postinoculation) were used for RNA-seq analysis. We carry comparison between similar materials at different times and between different materials at same times. The overlapping DEGs between the dominant phenotypes (L031 and F1 hybrid) and the recessive phenotype (Chancellor) were 1028 and 2214 DEGs, which were clearly lower than those between the dominant and recessive parents and thus could provide relatively accurate and valuable information. GO and KEGG enrichment analysis of DEGs revealed that other than the expected defense-related genes, differential up- and downregulation of genes from many other signaling networks were also involved. Comparative transcriptome analysis also revealed that early-stage postinoculation is important and suitable time points to study expression profiles and signaling pathways of resistance-related genes following fungal inoculation. qRT-PCR analyses showed highly consistent expression patterns of genes with RNA-seq data. The results will aid in the identification of genes and signaling pathways involved in powdery mildew response in wheat.

## 1. Introduction

Common wheat (*Triticum aestivum* L.) is one of the main staple foods for humankind, serving as a major source of carbohydrates and proteins. Among the many constraints to wheat production, powdery mildew (PM), caused by *Blumeria graminis* f. sp. *tritici* (*Bgt*), is an important biotrophic pathogenic wheat disease worldwide. It reduces kernel weight and damages grain quality, leading to yield losses of 10–30% [1]. Although agricultural chemicals also provide protection against PM, resistant cultivars are the most effective, economical, and environmentally sound strategy to protect the wheat crop.

At present, 60 resistance genes or alleles at 49 loci conferring resistance to PM (*Pm1*–*Pm53*, *Pm18* = *Pm1c*, *Pm22* = *Pm1e*, *Pm23* = *Pm4c*, and *Pm31* = *Pm21*) have been identified from different wheat sources [2, 3]. Of these genes, 31 were derived from common wheat and the remainder were derived, or putatively derived, from wild relatives. However, most genes become ineffective within a short period of use in agriculture because of changes in virulence or virulence frequency in pathogen populations [4].

Over the course of evolution, plants have developed sophisticated innate immune surveillance systems to perceive pathogen attack and to induce appropriate defense responses. Understanding the signaling pathways and plant responses after inoculation may help scientists uncover the molecular mechanisms of disease resistance and possibly allow resistance genes to be used in a more durable manner. Among the 49 reported *Pm* loci, only a few *Pm* resistance alleles have been cloned. For the PM resistance gene *Pm3*, 17 functional alleles (including *Pm3a-3g*) have been isolated and cloned. They share more than 97% nucleotide sequence identity and code for coiled-coil (CC), nucleotide-binding site, ARC1 and ARC2 (NB-ARC) and leucine-rich repeat (LRR) domain proteins [5–10]. *Pm8*, located on a 1BL.1RS translocation chromosome, is a PM resistance gene transferred to wheat from *Secale cereale* L. The gene was cloned based on sequence homology and is the rye ortholog of the wheat *Pm3* gene. Sequence analysis revealed that the PM8 and PM3B proteins share 81% sequence identity and that nucleotide diversity occurs mainly in solvent-exposed residues of the LRR domain [11].

In the recent years, genome-wide analysis of gene expression using RNA-seq has been widely implemented in several types of plants, especially in host-pathogen interaction.

In wheat, Xiao et al. [12] performed a transcriptome comparison of wheat landrace Wangshuibai and a mutant from Wangshuibai (namely, NAUH117) during infection by *Fusarium graminearum* and suggested that pathogen-related proteins, such as PR5, PR14, and the ABC transporter and JA signaling pathways, were crucial for Fusarium head blight (FHB) resistance, whereas the ethylene (ET) and reactive oxygen species/nitric oxide (ROS/NO) pathways were not activated in Wangshuibai and therefore might not be pivotal in defense against FHB. Using RNA-seq data from near-isogenic lines (NILs), harboring either the resistant or the susceptible allele for *Fhb1* and *Qfhs.ifa-5A*, Kugler et al. [13] investigated and found a gene coexpression network that was activated in response to *Fusarium graminearum*. In *Arabidopsis*, Zhu et al. [14] analyzed the dynamic defense transcriptome responding to *Fusarium oxysporum* infection using a strand-specific RNA-sequencing approach and identified many novel disease responsive genes, including noncoding RNAs. In addition, RNA-seq has been applied to other plant diseases, to investigate defense mechanisms against *Fusarium oxysporum* in banana [15] and white pine blister rust (WPBR, caused by *Cronartium ribicola*) in western white pine (*Pinus monticola*) [16]. Although RNA-seq has been widely used in many plants, few studies have been performed in wheat to determine transcriptomic changes in response to PM.

Although the PM resistance gene *Pm2* was identified several decades ago, it was still interested for resistance breeding because it confers resistance to many *Bgt* isolates and is effective and applied in cultivars. In the present work, the susceptible wheat cultivar Chancellor and resistant NIL L031 with resistance gene *Pm2* were used in RNA-seq analysis. To analyze the respective defense transcriptomes responsive to PM infection the resistant and susceptible wheat lines and to identify responsive genes at an early stage of infection, we compared the leaf transcriptomes of Chancellor, L031, and the F1 hybrid (resistant) at two time points (namely, 16 h and 96 h postinoculation, hpi). The reads were assembled de novo using Trinity platform software, and their abundances were determined to identify genes that were differentially expressed between infected leaves of the susceptible and resistant genotypes. We further identified and characterized some novel transcripts with differential abundance levels, which probably play roles in the host immune response of common wheat to *Bgt* infection.

## 2. Materials and Methods

### 2.1. Plant Material and Growth Conditions

Common wheat (*Triticum aestivum*) cultivar, Chancellor (susceptible), and its NIL L031 (resistant) with resistance gene *Pm2* were used in this study. They were obtained by crossing Ulka (donor of resistance gene) with Chancellor, and then backcrossed with Chancellor for 7 generations, and finally self-crossed and selected under *Bgt* inoculation [17]. They were provided by the Institute of Crop Science, Chinese Academy of Agricultural Sciences, Beijing. Chancellor was crossed with L031 to produce F1 hybrids that were also resistant to *Bgt* E09. Plants of the resistant F1 hybrid, Chancellor, and L031 were grown in growth chambers with a 16 h photoperiod and maintained at 18°C. The *Bgt* isolate E09 was used to infect seedlings at the three-leaf stage. Leaf samples collected from three plants at 16 and 96 hpi were separately pooled from three plants and immediately immersed in liquid nitrogen and stored at −80°C until further processing.

### 2.2. RNA Isolation and Quality Verification

Total RNAs were isolated from the mixed samples using a simple RNA extraction kit (Tiangen, Beijing, China) and treated with DNase I to remove any DNA contamination. The quality and integrity of the RNA were then verified by gel electrophoresis and a NanoPhotometer® spectrophotometer (IMPLEN, CA, USA). Beads with oligo(dT) were used to isolate poly(A) mRNA from total RNA.

### 2.3. RNA-seq Library Construction and Sequencing

Oligo(dT)-coated magnetic beads were used to enrich the mRNA (Qiagen GmbH, Hilden, Germany), which was then broken into fragments using fragmentation buffer. Using these cleaved mRNA fragments as templates, first- and second-strand cDNAs were synthesized. The double-stranded cDNA was further purified using a QiaQuick PCR extraction kit (Qiagen, Hilden, Germany), resolved for final reparation and poly(A) addition, and then connected using different sequencing adapters. The expressed sequence tag (EST) libraries were constructed by PCR amplification after checking the quality by agarose gel electrophoresis and sequencing using an Illumina HiSeq™ 2000 platform by Biomarker Technology Co. Ltd (Beijing).

### 2.4. Transcriptome De Novo Assembly

To obtain clean reads, raw reads, including reads with adaptors, reads in which unknown bases represented more than 5% of the total bases, and low-quality reads (percentage of low-quality bases with a quality value ≤ 5 in more than 50% of a read), were removed. The clean reads were then assembled de novo into longer contigs based on overlapping regions using the Trinity platform (http://trinityrnaseq.sourceforge.net/) [17]. Contigs from a different transcript and their distances were confirmed by mapping clean reads back to the corresponding contigs based on the paired-end information, and thus, the transcript sequences were determined. These sequences were defined as unigenes.

We have also attempted to use the genome sequence of *Triticum aestivum* (ftp://ftp.ensemblgenomes.org/pub/plants/release-34/fasta/triticum_aestivum/dna/) as a reference, but as the ratio of uniquely mapped reads by TopHat2 was lower than 70% for all samples, possibly reflecting the fragmented nature of the current genome release, we chose to use the de novo assembled transcriptome as a reference instead.

### 2.5. Annotation and Classification of Unigenes

Unigenes were annotated using BLASTX against various databases, including nr (NCBI nonredundant protein database), Swiss-Prot, Gene Ontology (GO), Kyoto Encyclopedia of Genes and Genomes (KEGG), and Clusters of Orthologous Groups (COG), using a cut-off *E* value of 10^−5^. Furthermore, unigenes were searched (using BLASTN) against the NCBI nucleotide database (nt) using a cut-off *E* value of 10^−5^. Assignment of unigenes to pathways was performed by searching the KEGG databases. The coding sequences of unigenes were determined based on the orthologous proteins. This analysis mapped all of the annotated unigenes to GO terms in the database and counted the number of unigenes associated with each term. TopGO software was then used to plot GO functional classification for the unigenes with GO term hits to view the distribution of gene functions of the species at the macro level.

### 2.6. Annotation of Expression

To evaluate the depth of coverage, all of the usable reads were realigned to each unitranscript using a Short Oligonucleotide Analysis Package (SOAP) aligner (http://soap.genomics.org.cn/soapaligner.html) and were then normalized into RPKM values (reads per kb per million reads) [18]. Differentially expressed gene (DEG) analysis was then performed using the Bioconductor package DESeq [19], which is based on the ratio of RPKM values. The false discovery rate (FDR) control method was used to identify the threshold of the *P* value in multiple tests to compute the significance of differences in transcript abundance. In the present analysis, only unitranscripts with absolute log_2_ ratios ≥ 1 and FDR significance scores < 0.01 were used for subsequent analyses.

Hierarchical clustering and heat map generation were performed in R. The gene expression data were log2-transformed and then quantile-normalized prior to generating the heat map for direct comparison of the data.

## 3. Validation of Gene Expression by Quantitative Real-Time PCR (qRT-PCR)

Gene-specific primers were designed using Primer Premier software (version 5.0) and the *Actin* gene was used as the internal control gene. The qRT-PCR reaction for target gene transcript amplification was carried out in a final volume of 25 *μ*L containing PCR buffer, 1 mM MgCl_2_, 0.2 mM dNTP, 1 *μ*L of SYBR green І, 1 U of Taq, 0.4 *μ*M of each forward and reverse primers, and 2 *μ*L diluted cDNA. The PCR reaction conditions were denaturation at 95°C for 5 min followed by 40 cycles of 95°C for 10 s, annealing at the appropriate temperature (from 57 to 61°C) for 30 s, extension at 72°C for 30 s, with a final extension at 72°C for 10 min. All reactions were done in triplicate. The amplification data were analyzed using iQ5 software version 1.0 (Bio-RAD, USA). The threshold cycle (Ct) values of the triplicate PCRs were averaged and relative quantification of the transcript levels was undertaken using the comparative Ct method. The ΔCt value of the calibrator (the sample with the highest ΔCt value) was subtracted from every other sample to produce the ΔΔCt value, and 2^−ΔΔCt^ was taken as the relative expression level for each sample.

## 4. Results

### 4.1. Transcriptome Sequencing by RNA-seq and De Novo Assembly

To identify differentially expressed genes in the susceptible *pm2pm2* genotype (Chancellor), resistant *Pm2Pm2* genotype (L031), and the resistant F1 hybrid (*Pm2pm2*) after inoculation with the *Bgt* isolate E09, leaf RNA samples of the three lines at two different time points (16 and 96 hpi) were prepared and sequenced using the Illumina sequencing platform. The 6 RNA-seq libraries included L031, F1, and Chancellor at 16 hpi (designated as T1, T2, and T3) and 96 hpi (designated as T4, T5, and T6). The global gene expression profiles at the first sampling were assumed to reflect early host responses triggered by PM infection, whereas the other profiles were assumed to more likely reflect a subsequent wider range of molecular pathways regulated by the host defense system.

After cleaning and checking the read quality, 4.16 G, 3.62 G, and 4.06 G of clean data were generated at 16 hpi, and 4.23 G, 3.47 G, and 4.15 G clean data were generated at 96 hpi (see Additional Table S1 available online at https://doi.org/10.1155/2017/7305684). Of the clean reads, more than 90% had read qualities of Q30 (0.1% sequencing error rate) or higher.

Reads were then assembled de novo using Trinity platform software, resulting in 316,787 transcripts and 112,033 unigenes with N50 lengths of 1742 bp and 1158 bp, respectively. Approximately 7.62% unigenes had lengths of more than 500 bp. These unigenes were annotated by Blastx alignment against the NCBI nr, Swissport, COG, GO, and KEGG databases. In total, 63,210 unigenes were annotated, and the percentage of aligned reads mapping to unigenes in the library was generally approximately 80%, which indicated an acceptable quality of the aligned reads.

### 4.2. RPKM Density Distribution of Transcript Profiling

**Figure 1:**
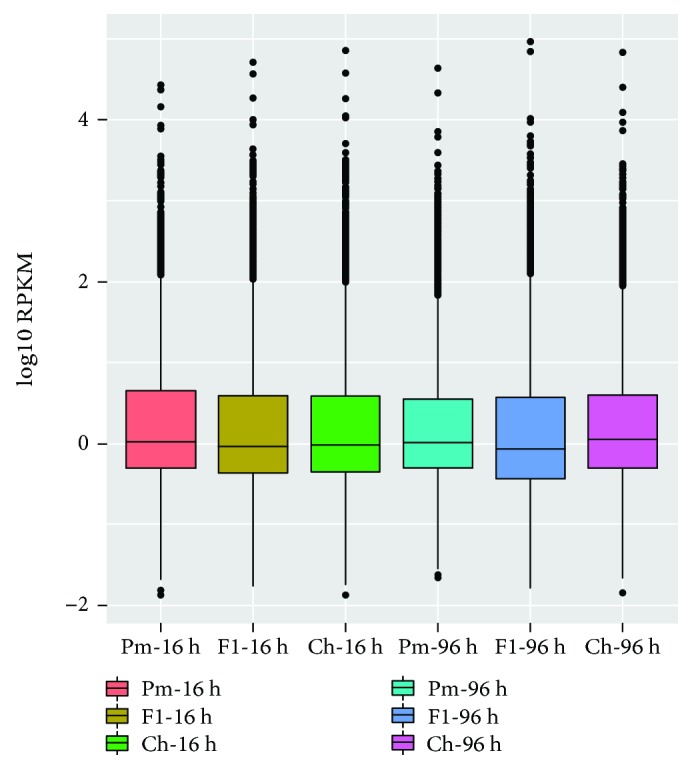
Box plots of relative log RPKM values based on all genes for each RNA-seq library. Differentially expressed genes between L031, F1, and Chancellor in response to Bgt isolate E09.

Normalized RPKM values were used to quantify the transcription levels in the reads, facilitating comparison of mRNA levels within and between samples [18]. The box plots of the relative log RPKM values for each RNA-seq library showed few distributional differences among the six libraries (Figure 1) suggesting the transcription profiles were similar.

### 4.3. Differentially Expressed Genes between L031, F1, and Chancellor in Response to Bgt Isolate E09

**Figure 2:**
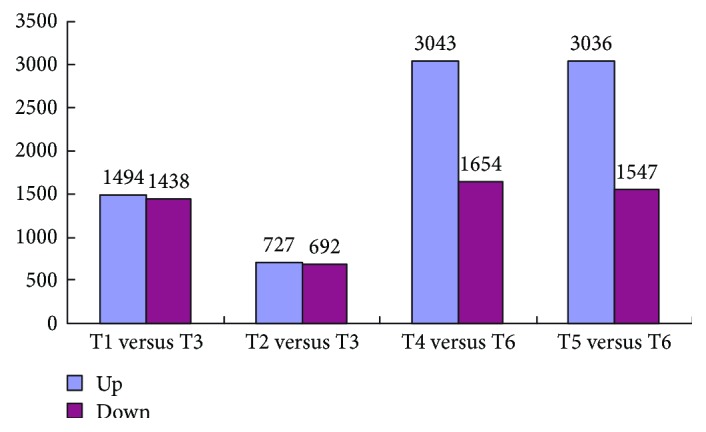
DEGs between L031, F1, and Chancellor at 16 and 96 hpi.

**Figure 3:**
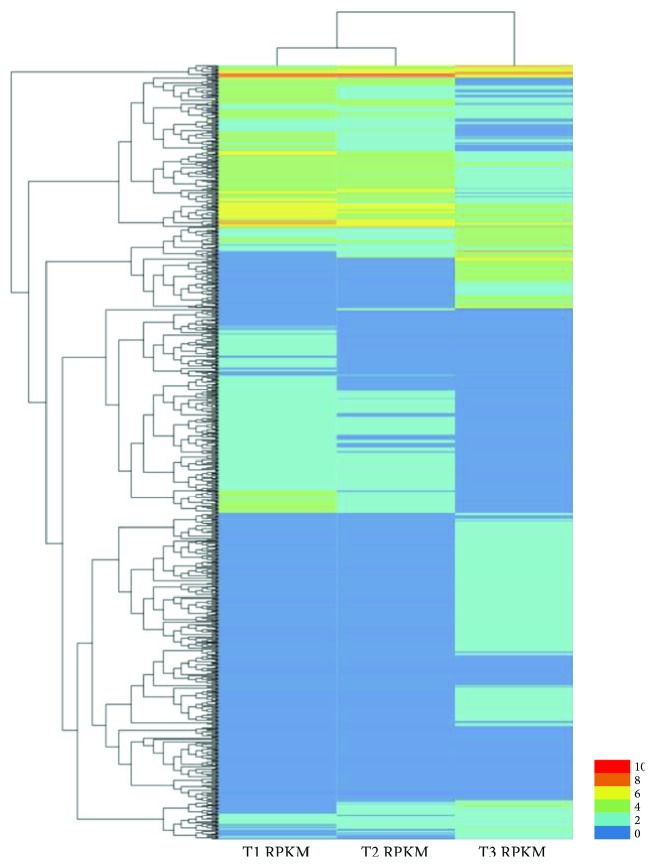
Hierarchical clustering of the 1028 coregulated DEGs between T1, T2, and T3.

Based on the RPKM data, putative DEGs were identified using an FDR of less than 0.01 and a fold change greater than 2. Additionally, up- or downregulated DEGs were also identified (here, up- or downregulation means that in combination A versus B, the gene expression level of B was higher or lower than that of A). Seven biologically meaningful pairwise comparisons were created for the DEGs (Table 1). Four pairwise comparisons between L031, F1, and Chancellor at different time points (T1 versus T3 and T2 versus T3 at 16 hpi and T4 versus T6 and T5 versus T6 at 96 hpi) and overlapping DEGs between T1 versus T3 and T2 versus T3 and between T4 versus T6 and T5 versus T6 were also characterized. Comparison between the L031 and Chancellor libraries at 16 hpi (T1 versus T3) revealed that 2932 genes were differentially expressed, of which 1419 were upregulated and 1438 downregulated (Figure 2). Similarly, 1419 DEGs were identified from comparison of the F1 and Chancellor (T2 versus T3) at 16 hpi, including 727 upregulated and 692 downregulated genes (Figure 2). In the longer period of disease development (96 hpi), 4697 (3043 upregulated and 1654 downregulated) and 4583 (3036 upregulated and 1547 downregulated) DEGs were identified in comparisons T4 versus T6 and T5 versus T6, respectively (Figure 2). Clearly, more DEGs were identified at 96 hpi than at 16 hpi, indicating that more downstream genes were affected in the later stages than in the early stages postinoculation. The results also inferred that the earlier stage postinoculation is more important for studying the expression and response of race-specific resistance genes after fungal attack, consistent with the results of Zhang et al. [20]. Hierarchical clustering analysis of the 1028 overlapping DEGs in Chancellor, L031, and the F1 hybrid was conducted (Figure 3), and the results revealed that L031 and the F1 were much more similar than either was to Chancellor.

**Figure 4:**
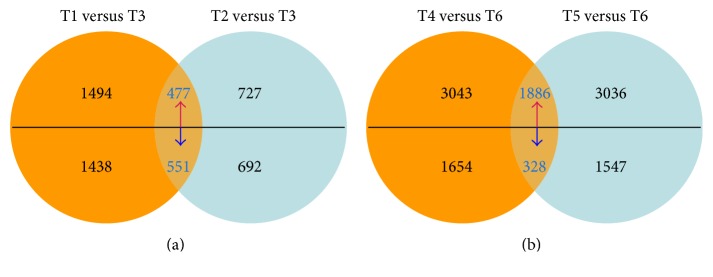
Venn diagram showing the coregulated DEGs between Chancellor, L031, and the F1 hybrid. Note: (a) coregulated DEGs between T1 versus T3 and T2 versus T3; (b) coregulated DEGs between T4 versus T6 and T5 versus T6; red and blue arrows indicate up- and downregulated DEGs, respectively. The Venn diagram was made using normalization data.

The Venn diagram of differentially coregulated genes revealed 1028 overlapping DEGs between “T1 versus T3” and “T2 versus T3,” among which 447 and 551 were up- and downregulated, respectively. At 96 hpi, 2214 coregulated DEGs were identified between T4 versus T6 compared to T5 versus T6, with 1886 and 328 genes were up- and downregulated, respectively. The overlapping DEGs were reduced from 2932 (T1 versus T3) and 4697 (T4 versus T6) to 1028 and 2214, and the numbers genes of interest were significantly reduced, especially at 16 hpi (Figure 4).

### 4.4. Coregulated Responses of Resistant and Susceptible Genotypes to Bgt Infection

**Figure 5:**
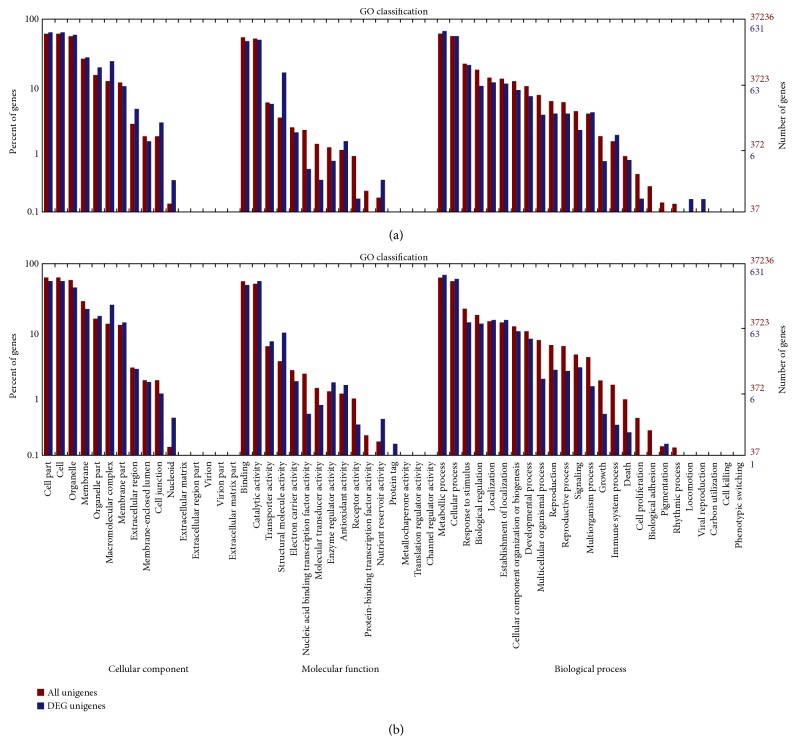
Gene enrichment analysis by GO term classifications of coregulated DEGs. (a) Coregulated DEGs between T1 versus T3 and T2 versus T3; (b) coregulated DEGs between T4 versus T6 and T5 versus T6. The results are summarized into three main categories: biological processes, cellular components, and molecular functions.

To determine their functions, the overlapping 1028 DEGs between (T1 versus T3) and (T2 versus T3) were mapped to classes in the GO database and were compared with the complete transcriptome background. These 1028 DEGs were categorized into 41 functional groups in the three main categories, “cellular components,” “molecular functions,” and “biological processes” (Figure 5(a). The “macromolecular complex” (146 unigenes) in the “cellular components” and “structural molecule activity” (96 unigenes) in the “molecular functions” categories were significantly enriched and exhibited the most significantly different expression levels between the resistant and susceptible genotypes.

**Figure 6:**
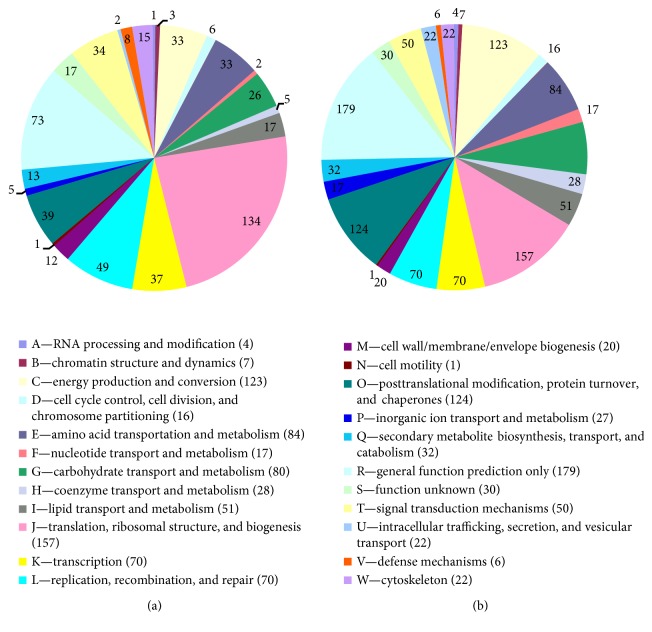
COG classifications of the coregulated DEGs. (a) Coregulated DEGs between T1 versus T3 and T2 versus T3; (b) coregulated DEGs between T4 versus T6 and T5 versus T6.

COG analyses identified 566 DEGs with annotations (Figure 6(a)). Approximately 94% the DEGs were classified in 14 major functional classes with 9 or more genes. In the J group, representing “translation, ribosomal structure, and biogenesis,” which consisted of 134 DEGs, most genes were related to the elongation factor, translation initiation factor, transferase, and 60S/40S ribosomal protein classes. Genes identified in “replication, recombination, and repair” (49 DEGs), “transcription” (37), “signal transduction mechanisms” (34), and “amino acid transport and metabolism” (34) comprised the other major groups.

**Figure 7:**
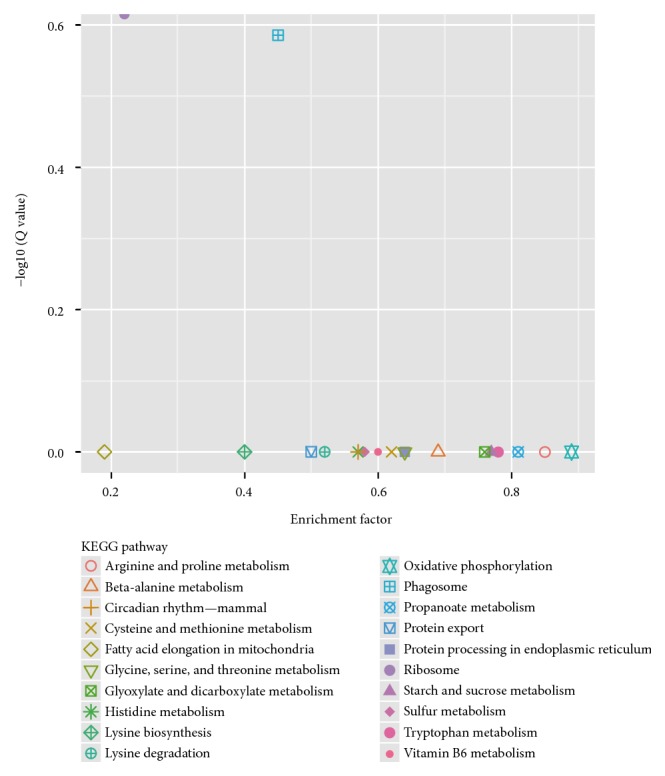
Distribution of the top 20 KEGG pathways from the KEGG database of the 1028 DEGs between T1 versus T3 and T2 versus T3.

**Figure 8:**
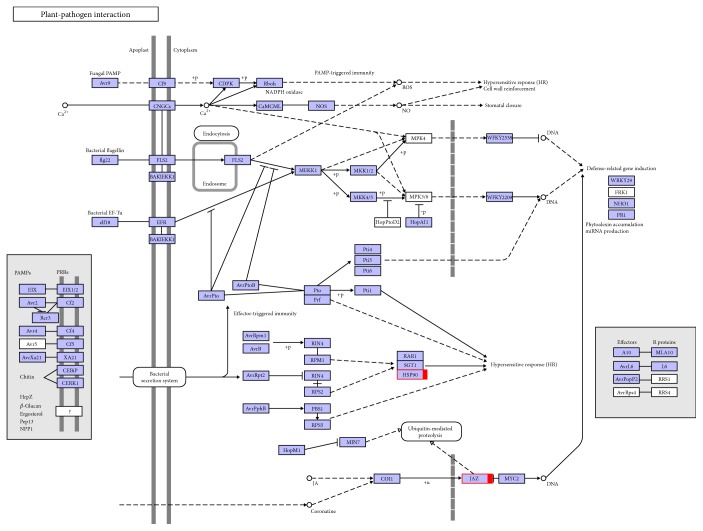
Unigenes matched with enzymes in plant-pathogen interaction pathways among the 1028 DEGs between T1 versus T3 and T2 versus T3. Note: DEGs encoding corresponding the enzyme in T3 were upregulated (red). The network diagram is from the KEGG website.

To further investigate the biochemical pathways of the DEGs, we mapped the 1028 DEGs to terms in the KEGG database and compared the results with the complete transcriptome background (Figure 7). Of the 1028 DEGs, 366 genes had KEGG Orthology (KO) IDs and could be categorized into 68 pathways. Figure 7 shows the 20 most enriched KEGG pathways. Of these, the “ribosome” and “phagosome” pathways were the most significantly enriched (corrected *P* value ≤ 0.05). In the “plant-pathogen interaction pathway,” three unigenes encoding the molecular chaperone heat shock protein (HSP90) functioning in the hypersensitive response (HR), K09487 (c100585.graph_c0; c155839.graph_c0), and K04079 (c124009.graph_c0) were identified (Figure 8). These unigenes were upregulated in Chancellor at 16 hpi. Novel proteins also interacted with the core HSP90 chaperone complex and regulated the accumulation and activation of nucleotide-binding LRR (NB-LRR) immune receptors. One JAZ unigene, K13464 (c87463.graph_c0), encoding protein TIFY 6B, a protein involved in the jasmonic acid (jasmonate, JA) signaling pathway, was also upregulated in Chancellor. This protein responds negatively as a repressor of jasmonate and is regulated by the proteasome in an SCF (COI1) E3 ubiquitin-protein ligase complex-dependent manner [21].

**Figure 9:**
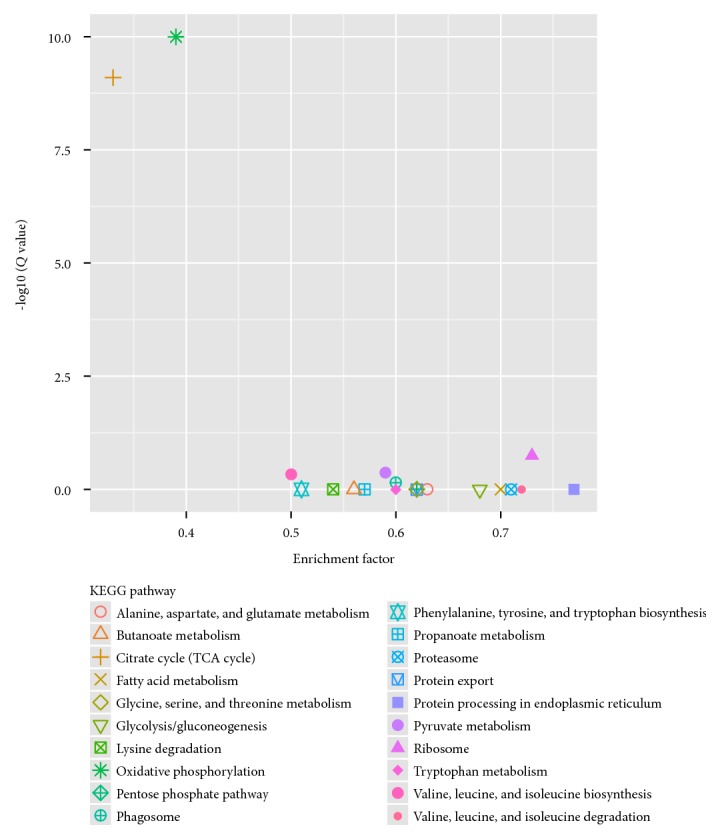
Distribution of top 20 KEGG pathways against the KEGG database of 2204 DEGs between T4 versus T6 and T5 versus T6.

**Figure 10:**
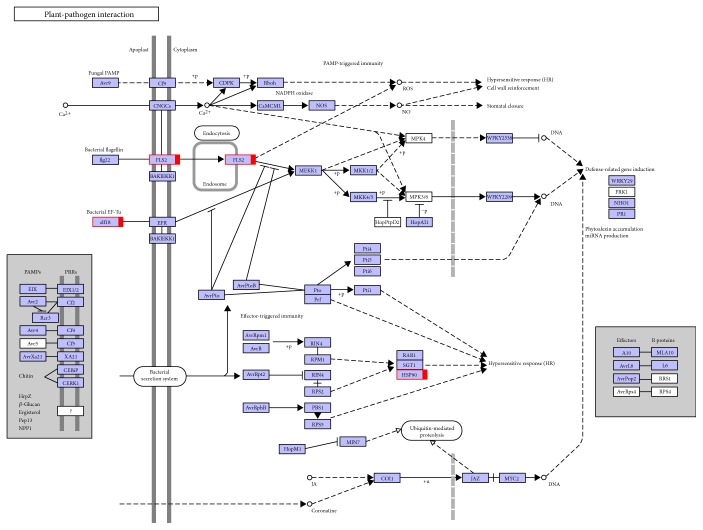
Unigenes matched with enzymes in plant-pathogen interaction pathways among 2204 DEGs between T4 versus T6 and T5 versus T6. Note: DEGs encoding the corresponding enzyme in T6 were upregulated (red). The network diagram is from the KEGG website.

GO, COG, and KEGG enrichment analyses were also conducted for the 2214 coregulated DEGs identified between T4 versus T6 and T5 versus T6. Similar to the results at 16 hpi, the functions “macromolecular complex” (305 unigenes) in the “cellular component” category and “structural molecule activity” (113 unigenes) in the “molecular function” category were also significantly enriched and exhibited the most significantly different expression levels between the resistant and susceptible genotypes (Figure 5(b)). COG analyses were also performed, and 1220 DEGs with annotations were identified (Figure 6(b)). The J group, representing “translation, ribosomal structure, and biogenesis,” consisted of 157 DEGs. The genes identified as belonging to “posttranslational modification, protein turnover, chaperones” (124), “energy production and conversion” (123), “amino acid transport and metabolism” (84), “carbohydrate transport and metabolism” (80), “replication, recombination, and repair” (70), and “transcription” (70) comprised the other major groups. KEGG enrichment showed that 1025 genes had KEGG Orthology (KO) IDs and were categorized into 101 pathways. Of these, “citrate cycle (TCA cycle)” and “oxidative phosphorylation” were the most significantly enriched pathways (Figure 9). At 96 hpi, the unigene c119685.graph_c0, K04079 encoding HSP90 was still upregulated in Chancellor (Figure 10). Two unigenes (c123807.graph_c0 and c136989.graph_c0), with KO ID (K13420) encoding flagellin-sensitive 2 (FLS2), and one unigene (c140501.graph_c0), with KO ID (K02358) encoding elongation factor Tu (EF-Tu) elf18, were also upregulated in Chancellor. FLS2 is a pattern-recognition receptor (PPR) that determines the specific perception of flagellin (flg22) [22], and EF-Tu represents a set of proteins that facilitate events involved in translational elongation. These proteins are both believed to be potent elicitors of defense response to pathogen-associated molecular patterns (PAMPs).

### 4.5. Genes Involved in Early and Later Signaling in Response to Bgt Infection

Three pairwise comparisons of different inoculation times (T1 versus T4, T2 versus T5, and T3 versus T6) were generated (Table 2) to identify dynamic defense processes involved in host-pathogen interaction.

**Figure 11:**
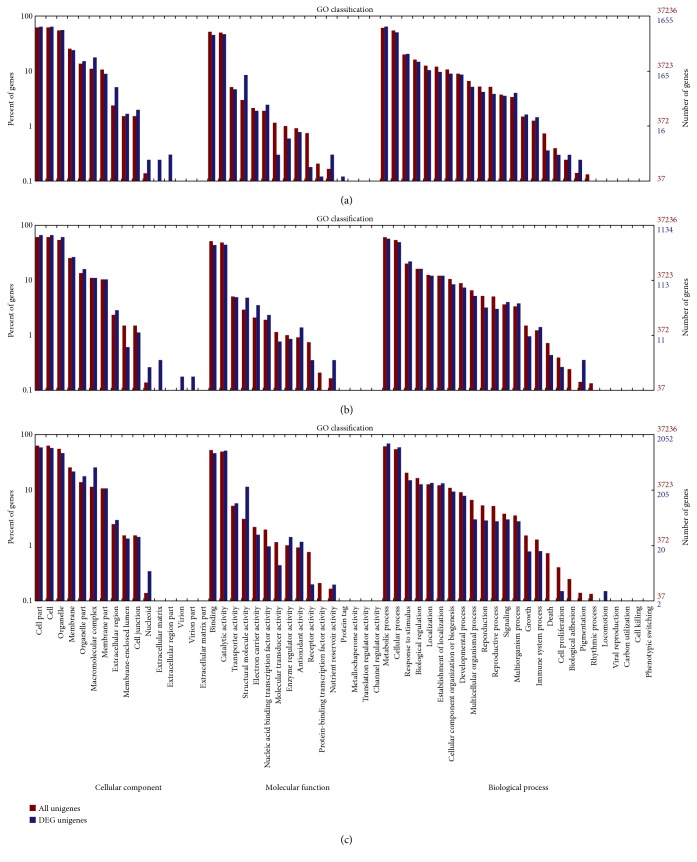
Gene enrichment analysis by GO term classifications of the coregulated DEGs. (a) DEGs in T1 versus T4; (b) DEGs in T2 versus T5; (c) DEGs in T3.

Comparisons between 16 and 96 hpi of the L031 libraries (T1 versus T4) showed that 2699 genes were differentially expressed, of which 1694 were upregulated and 1005 were downregulated at 96 hpi. Similarly, 1952 DEGs were identified from comparison between 16 and 96 hpi of the F1 hybrid (T2 versus T5), including 1376 upregulated and 575 downregulated genes. In the 16 versus 96 hpi comparison of the Chancellor libraries (T3 versus T6), 3682 DEGs were identified, of which 2950 genes were upregulated and 732 genes downregulated at 96 hpi. The results showed that more robust alterations in gene expression had occurred at the later stage of infection. These results indicated that the earlier stage postinoculation is a more suitable time point for studying gene expression of fungal resistance in wheat. As the leaves of Chancellor were attacked and damaged by infection, more DEGs were found than in the resistant L031 and the F1. GO, COG, and KEGG enrichment analyses were conducted for DEGs in the same genotype at different time points. Analysis of DEGs by GO analysis revealed that the functions “macromolecular complex” in the “cellular component” category and “structural molecule activity” in the “molecular function” category were significantly enriched and exhibited the most significantly different expression levels at different time points (Figure 11). The “extracellular matrix part” in the in the “cellular component” category and “pigmentation” in the “biological process” category showed different expression levels in L031 and the F1 hybrid, but not in Chancellor (Figure 11).

**Figure 12:**
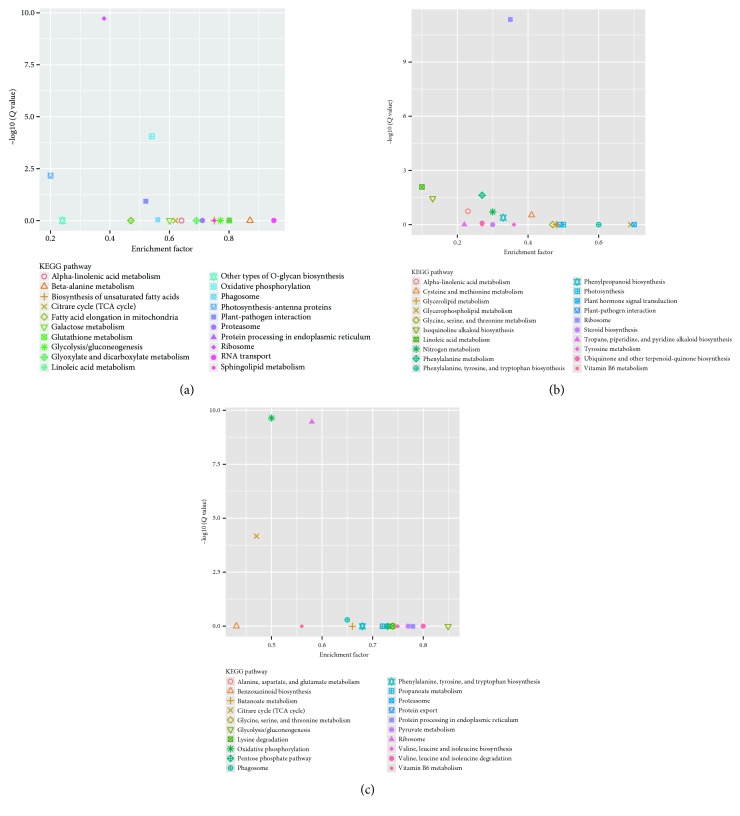
Distribution of the top 20 KEGG pathways in the KEGG database Note: (a) DEGs in T1 versus T4. (b) DEGs in T2 versus T5. (c) DEGs in T3 versus T6.

KEGG pathway analysis revealed that some enriched pathways of DEGs in L031 at different time points (T1 versus T4) were as follows: “ribosome,” “oxidative phosphorylation,” “RNA transport,” “protein processing in endoplasmic reticulum,” “phagosome,” “glycolysis/gluconeogenesis,” “proteasome,” and “citrate cycle (TCA cycle)” (Figure 12). The pathways enriched in Chancellor at 16 and 96 hpi (T3 versus T6) were similar to those between T1 versus T4. Longer-term infection increased the expression levels of “oxidative phosphorylation, TCA cycle, protein processing, and RNA transport” genes presumably to resist the fungal attack. Plant phagosomes also play important roles in plant defense.

The comparison of the F1 hybrid at different time points (T2 versus T5) indicated that other than the genes described in the KEGG pathway (Figure 12), “ribosome,” “cysteine and methionine metabolism,” “phenylalanine metabolism,” “plant-pathogen interaction,” “phenylpropanoid biosynthesis,” “nitrogen metabolism,” “plant hormone signal transduction,” and “glycerophospholipid metabolism” were also enriched. Phenylpropanoid metabolism leads to the biosynthesis of a wide array of phenylpropanoid secondary products, and the first step in this metabolic sequence involves the action of phenylalanine ammonia-lyase (PAL). After wheat or barley leaves are inoculated with their respective PM pathogens, PAL activity increases, indicating that changes in the metabolism of host phenolic compounds occur as a response to infection [23]. In the phenylalanine metabolism pathway of T2 versus T5, seven phenylalanine ammonia-lyases (c110027.graph_c0, c124467.graph_c0, c126780.graph_c0, c127926.graph_c0, c123754.graph_c1, c110377.graph_c0, and c118363.graph_c0) were upregulated at 96 hpi.

KEGG enrichment analysis also revealed that several pathways were specifically affected at the later stage of infection; these included secondary metabolic pathways such as “tropane, piperidine and pyridine alkaloid biosynthesis,” “steroid biosynthesis,” “ABC transporters,” “biotin metabolism,” “sphingolipid metabolism,” “glycosaminoglycan metabolism,” “folate biosynthesis,” “alpha-linolenic acid metabolism,” “regulation of autophagy,” “ubiquinone and other terpenoid-quinone biosyntheses,” and some “amino acid metabolism” pathways. Plant secondary metabolites exhibit several biological properties, such as defense mechanisms against herbivores, pests, and pathogens and stress tolerance, and serve as signal molecules or signal compounds in other biological functions.

### 4.6. Validation of DEG Expression by qRT-PCR

**Figure 13:**
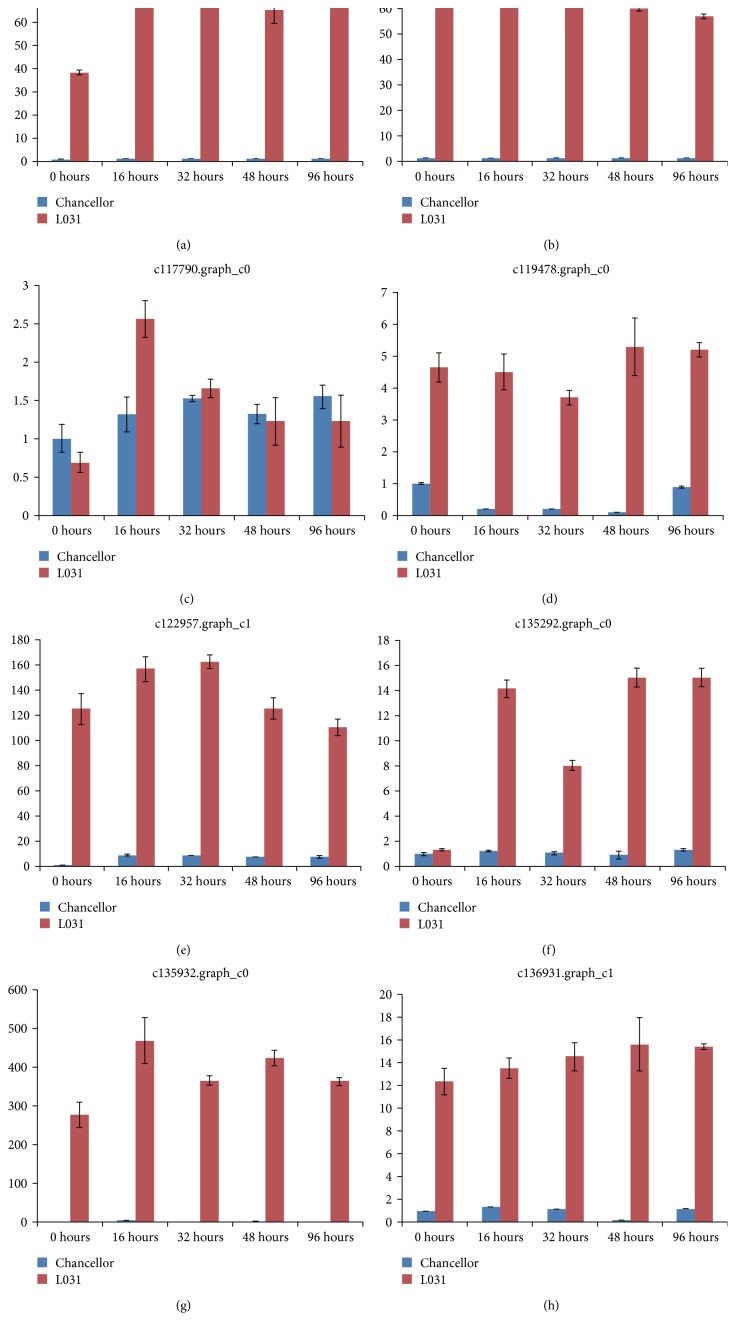
Validation of 8 DEGs in L031 and Chancellor using quantitative real-time RT-PCR (qRT-PCR). The *x*-axis shows the samples at different hours after inoculation with *Bgt*. E09.

R proteins were thought to recognize effectors in a “gene-for-gene” mode of resistance. We assumed that the R gene involved in reaction to *Bgt* would be more highly expressed in resistant than in susceptible genotypes. We therefore selected DEGs whose expression levels were zero or close to zero in the susceptible parent while having high RPKM values in L031 and the F1 hybrid at both time points. Some of the DEGs were well-known defense-related genes, such as LRR transmembrane protein kinases which triggering plant defense responses to pathogen and herbivore attacks. In this study, eight transcripts (c68959.graph_c0, c102478.graph_c1, c117790.graph_c0, c119478.graph_c0, c122957.graph_c1, c135292.graph_c0, c135932.graph_c0, and c136931.graph_c1) encoding the LRR family of receptor serine/threonine-protein kinases were selected for quantitative real-time PCR (qRT-PCR) analyses (Figure 13). The RPKM values of these transcripts were much lower (some were 0) in Chancellor; hence, these genes may be responsible for perception of fungal invasion and activation of downstream signaling cascades to induce defense responses. qRT-PCR analyses showed that the expression patterns of seven genes were consistent with the RNA-seq data, suggesting that our transcriptome analysis was accurate and reliable.

## 5. Discussion

### 5.1. Transcriptome Study about Wheat Reaction to PM

Transcriptome analysis is an important comprehensive and genome-wide tool for characterization and understanding the molecular basis of phenotypic variation in biology, including disease response. Analyzing the expression profiles of many genes simultaneously in large-scale expression studies, without making prior assumptions about candidate genes, allows the identification of new genes and molecular pathways associated with a particular trait. As the cost of sequencing decreases, RNA-seq is rapidly becoming an increasingly popular technique and an effective approach for transcriptome analysis. Unlike the detection capabilities of microarrays, which are limited by the microarray probes, RNA-seq offers the opportunity for de novo discovery and detection of novel transcripts, splice variants, and single-nucleotide variations, along with quantitative measurement of gene expression, enabling the discovery of novel coding and noncoding transcripts and fusion genes with both high-throughput and high-resolution capabilities [24–28].

Different approaches, including microarray analyses, have been employed to identify PM resistance genes in wheat. Xin et al. [29] compared leaf transcriptomes using Affymetrix wheat microarrays before and after *Bgt* inoculation of two wheat genotypes, namely, PM-susceptible cultivar Jingdong 8 and a NIL-carrying resistance gene *Pm30*. They found that PM resistance is a highly complex systematic response involving a large amount of gene regulation. Recently, Zhang et al. [20] analyzed the transcriptome of hexaploid wheat line N9134 inoculated with the Chinese *Pst* race CYR 3, compared to the same line inoculated with *Bgt* race E09 at 1, 2, and 3 days postinoculation to identify coregulated mRNAs that exhibited changes in expression patterns after inoculation with *Pst* or Bgt, and to identify mRNAs specific to fungal stress response. But few RNA-seq studies have been performed in wheat to determine transcriptomic changes in response to PM although RNA-seq has been widely used in many species.

### 5.2. Overlapping DEGs between Dominant Parent and F1 Hybrid with Recessive Parent Reduce Number of DEGs of Interest

The F1 hybrid has the heterozygous genotype (*Pm2pm2*), while Chancellor (*pm2pm2*) and L031 (*Pm2Pm2*) possess the homozygous genotype. The F1 exhibited the same phenotype (resistant) as the resistant parent, L031. Therefore, it might be predicted that the expression profiles of the hybrid and the affected downstream genes should be similar to those of L031. The differentially expressed transcripts between the F1 and Chancellor, especially the overlapping DEGs between L031 and the F1 hybrid compared to Chancellor, should be very useful and more accurate for identifying important genes involved in the host resistance conferred by *Pm2*.

Comparison of “T1 versus T3” revealed that 2932 genes were differentially expressed between the L031 and Chancellor libraries at 16 hpi and 1419 DEGs were identified from comparison of the F1 and Chancellor (T2 versus T3) at 16 hpi. Venn diagram revealed 1028 coregulated DEGs between “T1 versus T3” and “T2 versus T3.”

As a result of measurement noise or biological complexity, thousands of DEGs were identified between NILs and pairs of transgenic and nontransgenic lines, even in RNA-seq studies of monogenic traits [28]. Some DEGs also represented false-positive effects. However, the results indicated that the experimental design is accurate and efficient for identification of DEGs that are involved in signaling and defense pathways involved in PM response.

### 5.3. R Genes Involved in Pm2 Response to Bgt Isolate E09

Plants have developed sophisticated innate immune surveillance systems to perceive pathogen attack and to induce appropriate defense responses. One part of the system involves the induction of elicitors and effectors by membrane-bound receptors, which activate the putative PAMPs. Another branch of the immune monitoring system primarily uses intracellular resistance (R) proteins to recognize the presence of specific pathogen effector proteins in host cells [30–32]. Most R genes cloned to date encode NB-LRR proteins that recognize specific pathogen-derived products and initiate a resistance response that often includes a type of cell death, known as HR [33]. Sequence analysis has revealed that *Pm8* and *Pm3* belong to this family of NB-LRR type R genes. Following the perception of pathogen infection by R proteins, plants activate phytohormone metabolism and signaling pathways, including salicylic acid- (SA-) dependent signaling cascades and jasmonate and ethylene signaling pathways. Moreover, a wide range of additional pathways is also involved, including cell wall fortification, flavonoid biosynthesis, and metabolic processes. The detailed signaling cascades suggest that the induced defense responses involve multiple signal transduction pathways.

A unigene (c100703.graph_c0) presumed to encode a jasmonate-induced protein was identified and had representative sequences belonging to the well-known salicylic acid (SA) and/or jasmonic acid/ethylene (JA/ET) signaling pathways involved in plant defense response. Jasmonate is expressed widely in higher plants and plays an important role in wound and pathogen infection signal transduction pathways. It induces plants to activate the transcription of defense genes and to produce jasmonate-regulated proteins (JRPs). Transcripts belonging to the putative disease resistance protein RGA, RPP13 (c102287.graph_c1, c117921.graph_c0, c104233.graph_c0), cysteine-rich receptor-like protein kinase (c103786.graph_c3, c100842.graph_c0), and transcription initiation factor IIA subunit (c121687.graph_c0) were also identified.

Among the 1028 coregulated DEGs between T1 versus T3 and T2 versus T3, 119 DEGs with an RPKM value of 0 in T3 were identified. Meanwhile, 95 DEGs with an RPKM value of 0 in T6 were identified among 2204 overlapping DEGs between T4 versus T6 and T5 versus T6. Following the comparison of these 119 and 95 DEGs, 47 unigenes showing significant downregulation (RPKM = 0) in Chancellor at both time points were identified. These 47 genes and their putative functions annotated in the nonredundant (nr) and Swissprot databases are listed in Table S2.

These 47 genes correspond to disease resistance proteins RGA, RPM, and RPP; a few types of receptor-like protein kinases; and some hypothetical proteins. RGA2 and RGA4 belong to a disease resistance NB-LRR family containing 14 LRR (leucine-rich repeats) and 1 NB-ARC domains [34, 35]. RPM1 is an R protein that specifically recognizes avrRpm1 type III effector avirulence protein from *Pseudomonas syringae.* It also belongs to the disease resistance NB-LRR family and contains 4 LRR and 1 NB-ARC domains. It interacts with TIP49A, a protein known to interact with the TATA-binding protein complex (TBP), acts by interaction with RIN4, and probably triggers plant resistance when RIN4 is phosphorylated by avrRpm1 [36, 37]. RPP8 and the interaction with TCV-interacting protein (TIP) may be essential for the recognition of avirulence proteins and the triggering of defense response [38]. Receptor-like serine/threonine-protein kinases can also repress disease resistance signaling pathways triggered in response to bacterial pathogens, such as G-type lectin S-receptor-like serine/threonine-protein kinase in *Pseudomonas syringae* pv. *tomato*. Enhanced resistance to the virulent bacterial pathogen *P. syringae* pv. *tomato* is also accompanied by an increase in PR1 expression [39]. These unigenes are important for elucidating the molecular mechanisms of host resistance to *Bgt* infection in wheat.

## 6. Conclusion

In this study, a pair of wheat NILs differing by presence and absence of the *Pm2* resistance allele were used for RNA-seq after inoculation with *Bgt.* Comparative transcriptome analysis revealed that the earlier stage postinoculation is an important and suitable time point to study the gene expression profiles and signal pathways or resistance-related genes after fungal attack. Overlapping DEGs between the dominant phenotypes (L031 and F1 hybrid) and the recessive phenotype (Chancellor) significantly reduced the number of DEGs of interest, demonstrating that this method is useful and efficient for identifying of DEGs in studies of monogenic Mendelian traits. Some R genes and defense-related genes exhibiting significant expression profiling results between resistant and susceptible genotypes were also identified.

## Supplementary Material

Table S1. Quality of the RNA-seq data. 4.16 G, 3.62 G, and 4.06 G of clean data were generated at 16 hpi, and 4.23 G, 3.47 G, and 4.15 G clean data were generated at 96 hpi and more than 90% had read qualities of Q30 (0.1% sequencing error-rate) or higher in Table S1. Table S2. Forty seven co-regulated unigenes with RPKM values of 0 in Chancellor at two timepoints. Among the 1028 co-regulated DEGs between T1 versus T3 and T2 versus T3, 119 DEGs with an RPKM value of 0 in T3 were identified. Meanwhile, 95 DEGs with an RPKM value of 0 in T6 were identified among 2204 overlapping DEGs between T4 versus T6 and T5 versus T6. Following the comparison of these 119 and 95 DEGs, 47 unigenes in Chancellor at both time points were identified with an RPKM value of 0. 47 genes and their putative functions annotated in the non-redundant (nr) and Swissprot databases are listed in Table S2.

## Figures and Tables

**Table 1:** DEGs between different samples at the same time point.

Comparison	Number of DEGs	Upregulated DEGs	Downregulated DEGs
T1 versus T3	2932	1419	1438
T2 versus T3	1419	727	692
T4 versus T6	4697	3043	1654
T5 versus T6	4583	3036	1547
T1 versus T4	29,498	15,176	14,322
T2 versus T5	26,474	13,189	13,285
T3 versus T6	28,443	14,480	13,963

**Table 2:** Three pairwise comparisons of different inoculation times (T1 versus T4, T2 versus T5, and T3 versus T6).

Comparison	Number of DEGs	Upregulated DEGs	Downregulated DEGs
T1 versus T4	2699	1694	1005
T2 versus T5	1951	1376	575
T3 versus T6	3682	2950	732
